# Decrease in signal-related activity by visual training and repetitive visual stimulation

**DOI:** 10.1016/j.isci.2022.105492

**Published:** 2022-11-05

**Authors:** Andreas Marzoll, Kazuhisa Shibata, Taro Toyoizumi, Isha Chavva, Takeo Watanabe

**Affiliations:** 1 Department of Cognitive, Linguistic and Psychological Sciences, Brown University, Providence, RI 02912, USA; 2 Center for Brain Science, RIKEN, Wako, Saitama 351-0106, Japan

**Keywords:** Biological sciences, Neuroscience, Sensory neuroscience

## Abstract

While principles governing encoding mechanisms in visual perceptual learning (VPL) are well-known, findings regarding posttraining processing are still unrelated in terms of their underlying mechanisms. Here, we examined the effect of repetitive high-frequency visual stimulation (H-RVS) on VPL in an orientation detection task. Application of H-RVS after a single task session led to enhanced orientation detection performance (n = 12), but not in a sham condition (n = 12). If prior training-based VPL had been established by seven sessions in the detection task, H-RVS instead led to a performance impairment (n = 12). Both sham (n = 8) and low-frequency stimulation (L-RVS, n = 12) did not lead to a significant impairment. These findings may suggest reversal dynamics in which conditions of elevated network excitation lead to a decrease in a signal-related activity instead of a further increase. These reversal dynamics may represent a means to link various findings regarding posttraining processing.

## Highlights

• Visual stimulation leads to different behavioral changes depending on the learning stage
• Without prior learning, high-frequency stimulation leads to performance enhancement
• With prior learning, high-frequency stimulation leads to performance impairment
• Reversal dynamics may help explain disparate findings in post-training processing

## Graphical abstract

**Figure:**
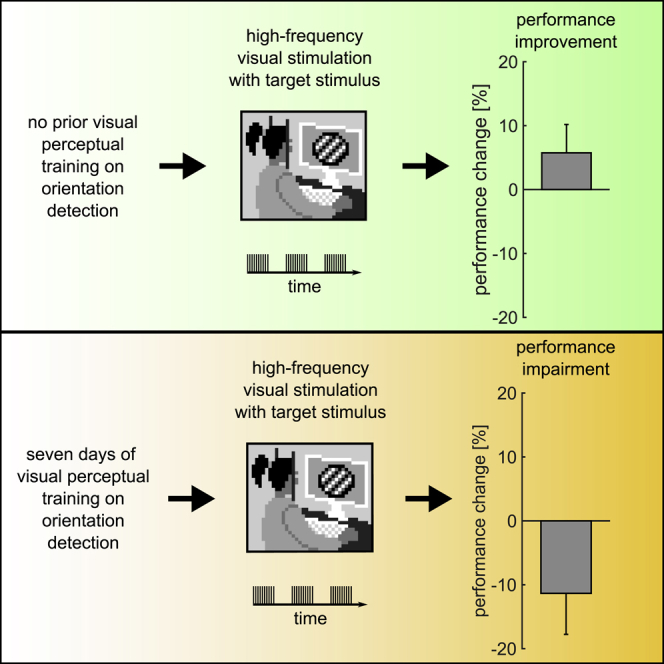

## Introduction

Visual perceptual learning (VPL) refers to long-term performance enhancement as a result of perceptual experience.^1^ Encoding mechanisms during VPL training have been extensively studied, and important principles have been identified.^1^^,^^2^^,^^3^ However, several research findings indicate that after initial training, several mechanisms continue to change memory through multiple stages, including stabilization/consolidation during wakefulness and reactivation followed by reconsolidation. As compared with research on encoding mechanisms, changes after training related to VPL, termed posttraining processing of VPL, remain enigmatic. Although there have been important findings regarding posttraining processing,^4^^,^^5^^,^^6^^,^^7^^,^^8^^,^^9^^,^^10^ many of these findings have been discussed and studied independently without being related to other findings based on their potentially common principles.

This failure may be due to two factors. First, the findings were not arrived at using the same measure as their dependent variable. For example, certain studies used behavioral performance,^5^^,^^6^^,^^7^^,^^8^^,^^9^^,^^10^ whereas others used the ratio of the concentration of excitatory neurotransmitters to the concentration of inhibitory neurotransmitters, termed the E/I ratio.^4^ Findings based on different measures as dependent variables cannot be easily linked to one another. Second, the various relevant studies used different and relatively complex training methods.^4^^,^^5^^,^^6^^,^^7^^,^^8^^,^^9^^,^^10^ This fact also makes it difficult to relate one finding to another.

This study used high-frequency repetitive visual stimulation (H-RVS), in which a visual stimulus intermittently flickers on and off at a relatively high frequency.^11^^,^^12^^,^^13^^,^^14^^,^^15^ The results of using this method could help relate our findings to those of other studies. H-RVS involves no task, which may prevent H-RVS results from being contaminated by task processing specific to each study and could be viewed as directly reflecting a common principle with respect to the different findings regarding posttraining processing.^16^

We conducted two experiments. In a preliminary experiment, H-RVS with an oriented Gabor stimulus was applied to a subject group that had not developed VPL through prior training in an orientation detection task that also employed Gabor stimuli (for details, see STAR methods). Consistent with previous studies,^14^^,^^15^^,^^17^ the subjects in the preliminary experiment exhibited a significant performance improvement due to H-RVS, while no such improvement occurred in a control group that did not receive H-RVS. In the main experiment, we applied H-RVS or low-frequency RVS (L-RVS) with an oriented Gabor stimulus, or no RVS, to each of the three subject groups that had already developed VPL as a result of repeatedly performing training sessions in an orientation detection task (training-based VPL) over 7 days. While performance levels in the posttest started out at similar levels in all three groups, performance quickly deteriorated in the H-RVS group and remained at a low level for the remainder of the session. Performance recovered by the following day. None of the L-RVS and no-RVS groups showed significant impairment.

These findings suggest reversal dynamics in which repetitive orientation detection performance and H-RVS, possibly after a period of elevated excitation, decrease activity related to stimulus orientations, in contrast to experimental conditions where no prior training-based VPL took place. Such reversal dynamics may help link important findings that were previously thought to be distinct.^4^^,^^5^^,^^6^^,^^7^^,^^8^^,^^9^^,^^10^

## Results

### High-frequency visual stimulation without prior training leads to performance enhancement

**Figure 1:**
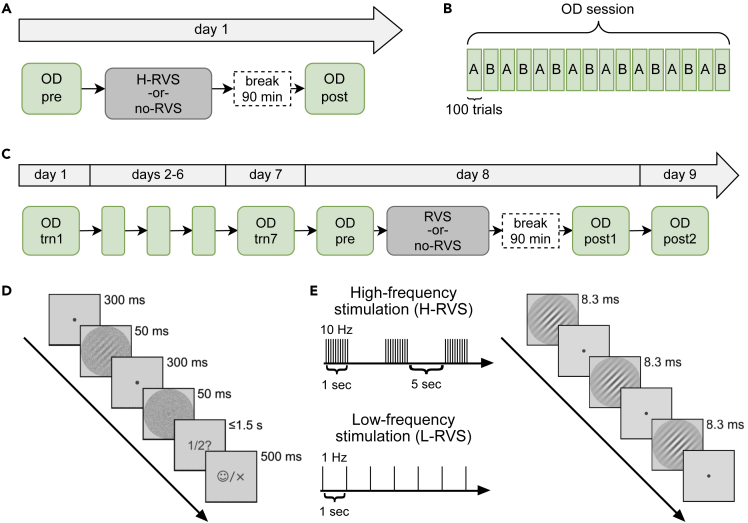
Experimental Setup (A) Procedure of the preliminary experiment. Either H-RVS or no RVS was applied after a single pretest session (pre) in the orientation discrimination (OD) task. OD performance was again evaluated in a posttest session (post) after a break of 90 min. (B) Block structure of an orientation discrimination session (one block = 100 trials). The two orientations (*A* and *B*) were presented in an alternating block-wise fashion. (C) Procedure of the main experiment. During the first seven days (trn1 – trn7) of the experiment, subjects performed training sessions in the OD task. Subjects were randomly assigned to one of the H-RVS, L-RVS, and no-RVS groups. On day 8, performance 90 min after (post1) the cessation of RVS and one day after (post2) the cessation of RVS was compared to performance immediately before RVS (pre). (D) Stimulus sequence during a two-interval-forced-choice OD task. (E) Left: Temporal patterns of H-RVS and L-RVS. Right: Stimulus sequence during RVS. Stimulus onset asynchronies (SOA) was 100 ms for H-RVS (intra-train) and 1000 ms for L-RVS. The stimulated orientations were counter-balanced across subjects and groups. Subjects in the no-RVS group were presented with a fixation point without exposure to flickering Gabor stimuli (for details, see STAR methods).

A number of studies have shown that H-RVS leads to VPL without prior task training.^14^^,^^15^^,^^17^ However, the experimental procedures in these studies are significantly different from those of the main experiment of this study. Thus, we conducted a preliminary experiment (Figure 1A; n = 24) to test whether a performance enhancement would be observed with the same procedures as used in the main experiment, except that no prior training was conducted. Twelve subjects performed an orientation detection task (Figure 1D) on two orientations (A and B) in alternating blocks (Figure 1B) before (pretest) and after (posttest) H-RVS. To test whether a performance enhancement, if any, is due to a test effect in which the pretest contributes to the performance enhancement, a different group of 12 subjects was asked to conduct only the pretest and posttest without any H-RVS with otherwise identical procedures.

**Figure 2:**
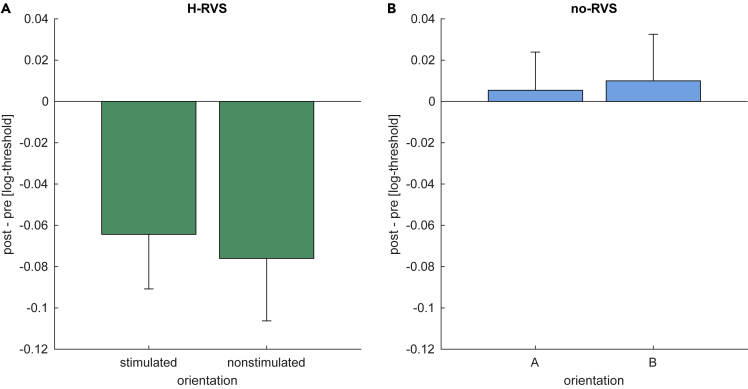
H-RVS without preceding training-based VPL leads to performance improvements Log-threshold differences between pre and post-sessions. Summary statistics are shown as means ± SEM. (A) In the H-RVS group, H-RVS was applied between the pre and post-session. Performance changes for both the stimulated and the nonstimulated orientation are shown. (B) Performance changes when no RVS was applied between pre and post-sessions. Results for orientations *A* and *B* are shown, which corresponded to the same orientations as in the H-RVS group.

Following H-RVS (Figure 1E) with one of these orientations (either A or B, counter-balanced across subjects), a significant improvement in orientation detection ability was observed for both orientations (Figure 2A). The improvement cannot be accounted for by a retest effect (Figure 2B).

The results of the subsequent statistical analysis confirmed this observation. A 3-way mixed-design ANOVA with within-subject factors of session (pretest vs. posttest) and orientation (stimulated vs. nonstimulated) and the between-subject factor group (presence vs. absence of H-RVS) revealed no significant main effects of either orientation (*F*_1,22_ = 0.73, p = 0.40), session (*F*_1,22_ = 3.67, p = 0.068) or group (*F*_1,22_ = 1.82, p = 0.19). There was no significant orientation × session × group interaction (*F*_1,22_ = 0.39, p = 0.54), which indicates that the effect of H-RVS was not specific to the stimulated orientation. However, there was a significant session × group interaction (*F*_1,22_ = 5.70, p = 0.026, $\eta_{p}^{2}$= 0.21). Post-hoc paired *t* tests with Bonferroni-Holm correction reveal a significant difference between sessions in the H-RVS group (*t*_11_ = 3.04, p = 0.036, Cohen’SD = −0.62), but no significant difference in the no-RVS group (*t*_11_ = −0.33, p = 1.00). These results indicate that H-RVS in the current paradigm led to performance enhancement.

### High-frequency visual stimulation after prior training leads to performance impairment

We recruited 32 subjects, who were randomly assigned to one of the three RVS groups: H-RVS (n = 12), L-RVS (n = 12), and no RVS (n = 8). Before receiving RVS, all subjects completed seven training sessions (on separate days) in an orientation detection task (Figures 1B–1D). For each subject, two orientations (A and B) were trained in alternating blocks. On day eight, the subjects were asked to perform the task in a pretest session. Then, subjects from the H-RVS and L-RVS groups received 1 h of RVS with either orientation A or B and whose flicker frequency depended on the group (Figure 1E). Subjects in the no-RVS group were asked to maintain their gaze at the fixation point presented at the center of the display while no RVS was being applied to them. In all groups, subjects had to perform a central oddball task to ensure their continued fixation (see STAR methods). Ninety minutes after the cessation of RVS in the H-RVS and L-RVS groups and the cessation of fixation in the no-RVS group, the subjects were asked to perform another session of the orientation detection task (“post1”). We only analyzed the first 16 blocks here (see STAR methods). To test the long-term effect of RVS, a final session of the orientation detection task was performed on the following day (“post2”).

**Figure 3:**
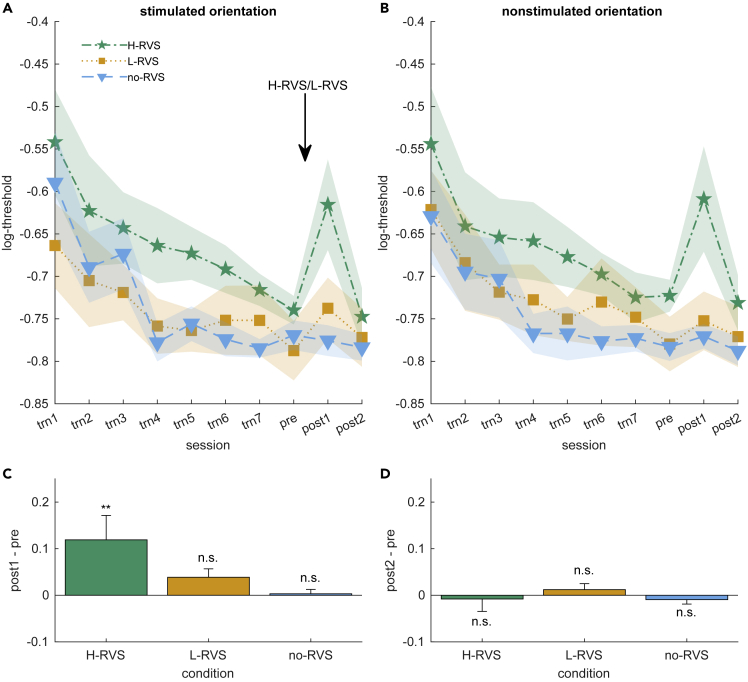
H-RVS after training-based VPL leads to a transient, orientation-nonspecific impairment in orientation detection performance Summary statistics are shown as means ± SEM. (A and B) Sessionwise signal-to-noise thresholds for the stimulated (A) and nonstimulated orientations (B). After VPL (trn1 – trn7), a strong and orientation-nonspecific impairment between the pre-RVS and post1 sessions occurred after H-RVS (arrow), which returns to pre-RVS levels on the following day (post2). (C and D) Mean changes in signal-to-noise thresholds from pre to post1 (C) and from pre to post2 (D) across the two orientations for each group. (∗∗) indicates p < .01, after Bonferroni-Holm correction.

VPL as a result of repetitive performance on the task for seven training days occurred with both target orientations in all three groups (Figures 3A and 3B; trn1-trn7). This observation was confirmed by the results of a 3-way mixed-design ANOVA on the within-subject factors of session (trn1-7) and orientation (stimulated vs. nonstimulated) and on the between-subject factor of group (H-RVS vs. L-RVS vs. no-RVS). There was a significant main effect of session (*F*_2.7,78.4_ = 12.0, p < .001, $\eta_{p}^{2}$ = .29, Greenhouse-Geisser corrected with ε = .45) but not of group (*F*_2,29_ = 1.26, p = .30, $\eta_{p}^{2}$ = .08) or orientation (*F*_1,29_ = 0.06, p = .81, $\eta_{p}^{2}$ = .002). There was a significant orientation × group interaction (*F*_2,29_ = 4.19, p = .025, $\eta_{p}^{2}$= .22), although subsequent Bonferroni-Holm-corrected post-hoc tests could not pinpoint specific significant comparisons (all *p*s > .21).

A large impairment in orientation detection ability was observed following H-RVS but not following L-RVS or no-RVS (Figures 3A–3C; post1). On the following day, the impaired performance after H-RVS recovered to pretest levels (Figure 3D; post2). The following statistical analysis confirmed the preceding observations. We performed a 3-way mixed-design ANOVA on the within-subject factors of session (pre, post1, vs. post2) and orientation (stimulated vs. nonstimulated) and on the between-subject factor of group (H-RVS vs. L-RVS vs. no-RVS). There was a significant main effect of session (*F*_1.41,40.89_ = 6.87, p = .006, $\eta_{p}^{2}$= 0.19, Greenhouse-Geisser corrected with ε = .71). Importantly, there was a significant effect of session × group interaction (*F*_2.82,40.89_ = 3.03, p = .043, $\eta_{p}^{2}$ = 0.17, Greenhouse-Geisser corrected with ε = .71). This result indicates that changes in performance were dependent on the type of RVS. There was no significant effect of session × group × orientation interaction, indicating that the effect was not specific to orientation (*F*_4,58_ = 0.35, p = .76). Because of this lack of evidence for orientation-specific effects, subsequent analyses excluded this factor of orientation. Levene’s test indicated that the assumption of variance homogeneity was violated in the post1 session of the H-RVS group for one orientation (stimulated orientation: *F*_2,29_ = 2.56, p = .01; nonstimulated orientation: *F*_2,29_ = 5.39; p = .095). This outcome was due to high interindividual variation in susceptibility to performance impairment. However, because the sample sizes of the groups are reasonably balanced, ANOVA can be considered robust to this violation.^18^ Post-hoc paired *t* tests with Bonferroni-Holm correction for the difference between pretest and post1 indicated that this impairment (Figure 3C) was significant in the H-RVS group (*t*_11_ = −4.37, p = 0.002, Cohen’s *d* = 0.77) but not in the L-RVS (*t*_11_ = −1.42, p = 1.00) or no-RVS group (*t*_7_ = −0.09, p = 1.0). The impairment in the H-RVS group did not persist to the next day (Figure 3D), as corroborated by a lack of significant differences from pretest to post2 in the H-RVS (*t*_11_ = 0.30, p = 1.0), the L-RVS (*t*_11_ = −0.44, p = 1.0), and no-RVS (*t*_7_ = 0.29, p = 1.0) groups.

Small performance reductions were also observed at post1 in the L-RVS and no-RVS groups (Figure 3C). Since no orientation stimulus was presented to the no-RVS group, the reduction may be accounted for by a general state that occurred across the three groups. However, *t* tests indicated a significant performance difference between the H-RVS and no-RVS groups at post1 (*t*_18_ = 2.23, p = .039, Cohen’s *d* = 1.02), whereas no significant difference was found between the L-RVS and no-RVS groups (*t*_18_ = 0.62, p = .53). These results indicate that performance impairment due to H-RVS in fact occurred, whereas no significant change in performance due to L-RVS occurred. Also, see the supplemental information for further analysis results which excluded the possibility that the impairments in the H-RVS group were related to individual differences prior to RVS.

Although group-level differences did not reach significance in the ANOVA, the possibility remains that the impairments observed in the H-RVS group were related to individual differences prior to H-RVS. We investigated the following four possibilities through a mediation analysis. As shown later in discussion, none of these possibilities turned out to have a significant influence on the performance changes after RVS/no-RVS.

1. Effect of pretraining performance: It is possible that impairment merely reflected a return to the pretraining performance levels observed on Day 1 (Figures 3A and 3B; trn1). Because the H-RVS group exhibited slightly worse performance than the other groups on Day 1, this may explain—at least partly—the larger effect size of the impairment after RVS.
2. Effect of training-based VPL: The amount of training-based VPL could explain the observed effect. We defined the amount of training-based VPL as the percent improvement between training sessions 1 and 7 [i.e., $\frac{trn7-trn1}{trn1}$].
3. Effect of the performance in the pretest session: The H-RVS group displayed somewhat worse performance in the session immediately preceding the intervention (pre) than the other two groups, which could be related to the lower performance after RVS.
4. Posttraining learning curve slope: Lower performance in the pretest and H-RVS groups could indicate that these subjects had not yet reached asymptotic performance, while the subjects from the other two groups had reached an asymptote in performance and thus differed in the learning stage. To test this possibility, we fitted learning curves (for details, see STAR methods) to the training performance from Day 1 to Day 7 (Figures 3A and 3B; trn1-trn7). The slope measured on Day 7 of these functions can be taken as an indicator of how settled at their performance level the subjects were. A slope close to zero indicates asymptotic performance, which was the case for most subjects.

**Figure 4:**
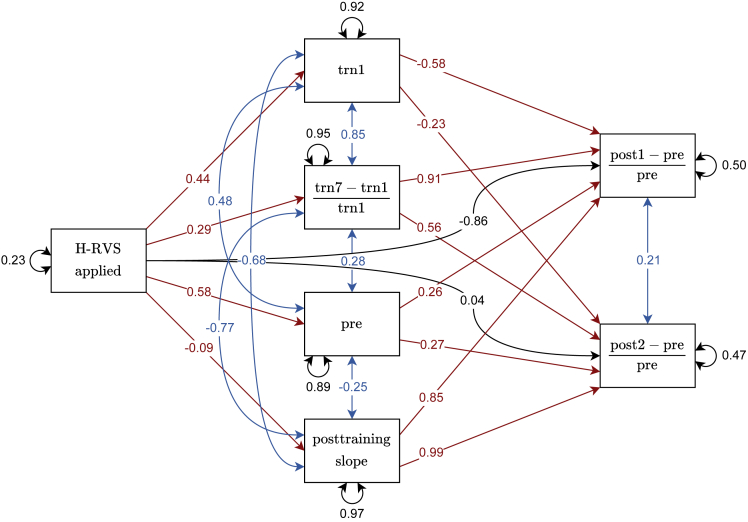
Path plot of the mediation analysis Numbers on the arrows indicate standardized coefficients estimated by the model. Black arrows indicate the direct effects of H-RVS on percent changes in orientation detection performance in the post1 and post2 session [$\frac{post1-pre}{pre}$ and $\frac{post2-pre}{pre}$]. Red arrows indicate the indirect effects via mediating variables (trn1: pretraining performance, $\frac{trn7-trn1}{trn1}$: training-based performance improvement [percent], pre: performance in the pre-stimulation session, posttraining slope: modeled learning curve slope at the end of the training phase). Blue arrows indicate residual covariances. Numbers next to recurrent arrows indicate residuals.

In the mediation analysis (Figure 4), we attempted to predict the percent changes between the pretest session and the two post-sessions [i.e., $\frac{post1-pre}{pre}$ and $\frac{post2-pre}{pre}$] from the presence or absence of H-RVS stimulation. The four variables mentioned above were added as mediators to the model to check if they can partially explain the observed impairment in the H-RVS condition. A significant mediation might indicate a confounding effect of these variables on the behavioral results. Consistent with our previous analysis, H-RVS significantly predicts performance changes in the post1 session (β = −0.79, z = −2.37, p = .018), but not in the post2 session (β = 0.16, z = 0.46, p = .65). An analysis of the indirect effects shows that none of the four variables significantly mediates these changes. This was the case for the changes in the post1 session (pretraining performance: β = −0.26, z = −0.94, p = .35; training-based VPL: β = 0.26, z = 0.76, p = .45; pretest performance: β = 0.15, z = 1.08, p = .28; posttraining learning curve slope: β = −0.08, z = −0.25, p = .80) and in the post2 session (pretraining performance: β = −0.10, z = −0.52, p = .61; training-based VPL: β = 0.16, z = 0.70, p = .48; pretest performance: β = 0.16, z = 1.13, p = .26; posttraining learning curve slope: β = −0.09, z = −0.25, p = .80). The direct effects of H-RVS on performance changes were significant in the case of post1 (β = −0.86, z = −3.16, p = .002) but not post2 (β = 0.04, z = 0.14, p = .89). Consequently, the direct effect of H-RVS on performance changes in post1 accounts for 91.8% of the explained variance in this model. This suggests that the four potentially confounding variables had a negligible effect on the observed pattern of results.

### The effects of high-frequency high-frequency visual stimulation require a period of build-up to occur

**Figure 5:**
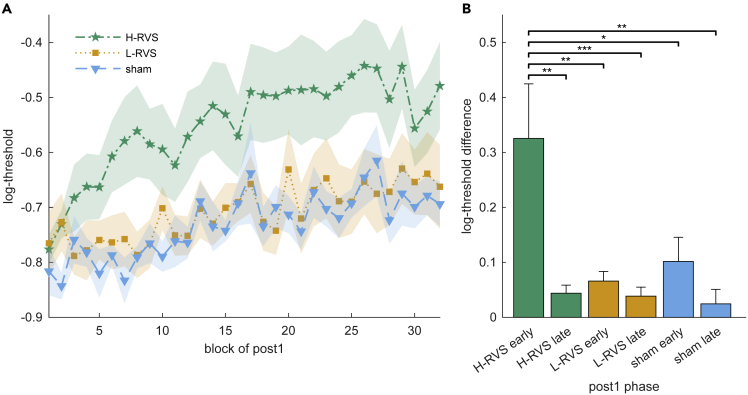
The effects of high-frequency RVS require a period of build-up to occur Summary statistics are shown as Mean ± SEM. (A) Block-wise signal-to-noise thresholds in post1, from 90 min until about 180 min after the stimulation offset for both orientations combined. (B) Comparison of performance changes from individually fitted power functions for blocks 1–10 (early) and 23–32 (late). Early impairment in the H-RVS condition was significantly larger than in all other conditions and phases. (∗) indicates p < .05, (∗∗) indicates p < .01, (∗∗∗) indicates p < .001.

In order to track the time course of the same-day effects after H-RVS (i.e. during post1), we looked at the block-wise performance of all 32 blocks in the orientation detection task (Figure 5A). This session spans the time from 90 min until about 180 min after the offset of RVS. Rather than a strong initial effect in the H-RVS group, the opposite was the case. This group started at a similar performance level to the others (this counts against the possibility that a relatively lower performance with this group before H-RVS caused a lower performance after H-RVS). However, during the first few blocks, performance decreases rapidly. Furthermore, there is no sign of attenuation in performance decreases until the end of the session. We quantified these observations by fitting power functions to individual block-wise performances and comparing estimated performance changes for the first and last 10 blocks of the session (Figure 5B). A mixed-design ANOVA with within-subject factor phase (early vs. late) and between-subject factor group (H-RVS vs. L-RVS vs. no-RVS) revealed significant main effects of phase (*F*_1,29_ = 9.07, p = .005, $\eta_{p}^{2}$= .24) and group (*F*_2,29_ = 4.90, p = .015, $\eta_{p}^{2}$= .25). Importantly, there is a significant phase × group interaction (*F*_2,29_ = 3.76, p = .035, $\eta_{p}^{2}$= .21). Subsequent Bonferroni-Holm-corrected post-hoc *t* tests show that the early change in the H-RVS group is significantly larger than in all other combinations of phase and group (vs H-RVS-late: *t*_11_ = 4.11, p = .004, Cohen’s *d* = 0.73; vs L-RVS-early: *t*_22_ = 3.89, p = .003, Cohen’s *d* = 0.69; vs L-RVS-late: *t*_22_ = 4.30, p < .001, Cohen’s *d* = 0.76; vs sham-early: *t*_18_ = 3.00, p = .044, Cohen’s *d* = 0.53; vs sham-late: *t*_18_ = 4.03, p = .002, Cohen’s *d* = 0.71). There were no significant differences between other combinations of phase and group (all absolute *t* values <0.92, *p*s = 1.0). These results are robust to moderate changes to the number of included blocks.

## Discussion

In this study, we applied H-RVS to a group of subjects after training-based VPL was established through seven days of training. At the beginning of the posttest, in which the same orientation detection task as in the VPL training stage was performed 90 min after RVS, no effect of H-RVS was observed. However, as progressively more test trials were conducted, performance decreased rather than increased. The performance impairment disappeared on the following day. In contrast, H-RVS with an untrained subject group that was otherwise subjected to the same procedures induced a significant performance improvement.

Performance changes (i.e., impairment or enhancement) after H-RVS depended on whether training-based VPL had been established before H-RVS. This outcome indicates that the performance impairment cannot be entirely accounted for by general fatigue or other unspecific effects.

Among the three groups of subjects who had established training-based VPL, the mean performances on the first day of training and in the pretest of the H-RVS group were lower to some degree, but not significantly, lower than those of the L-RVS and no-RVS groups. However, our statistical analysis indicates that these differences cannot account for the impairment after H-RVS. Moreover, at the beginning of the posttest, there was no significant difference in the performance among the three groups of subjects. Therefore, it is unlikely that the lower performance in the H-RVS group on the first day of training or in the pretest of the H-RVS group can account for the performance impairment in the H-RVS group.

Our findings may be regarded as a manifestation of interesting network activity changes related to visual signal processing. Both training-based VPL and performance enhancement by high-frequency sensory stimulation are each associated with increased levels of cortical excitation in involved sensory networks.^19^^,^^20^^,^^21^ In the case of H-RVS without prior training-based VPL, this increased cortical excitation may lead to stronger signal-related activity in the related visual areas, leading to increased performance by facilitating offline gains (cf.^19^^,^^22^). From this viewpoint, our findings with H-RVS after prior training-based VPL can be understood as evidence of reversal dynamics. Within these dynamics, conditions of elevated network excitation, possibly beyond the level of either training-based VPL or high-frequency stimulation individually, lead to a net decrease in a signal-related activity instead of a further increase, as is the case after either intervention alone. Such “paradoxical” effects on excitation, measured by phosphene thresholds, have been observed after high-frequency repetitive transcranial magnetic stimulation (rTMS) in conditions of abnormally elevated baseline excitation.^20^^,^^23^^,^^24^^,^^25^^,^^26^ Given that the results from stimulation using different paradigms (H-RVS vs. high-frequency rTMS) on significantly different measures (behavioral performance vs. phosphene thresholds) are in accordance with the reversal dynamics, the reversal dynamics of signal-related activity could be a broadly applicable principle.

Related alternative explanations, that could also lead to the observed decrease in signal-related activity, are an increase of internal noise in the affected sensory systems, which would cause diminished performance through a hampered readout of sensory signals by high-level decision-making areas.^27^^,^^28^ Furthermore, combined training-based VPL and H-RVS could cause sensory neurons to reach the limits of their dynamic firing range in a form of response saturation due to overexcitation, which would cause them to lose sensitivity. On the flip side, H-RVS without prior training-based VPL could have led to increased signal-related activity by increasing the signal strength itself or reducing internal noise.

Whatever the exact neural mechanism behind these findings may be, it is apparent that the underlying mechanism of VPL consolidation before and after long periods of training are different, which has earlier been suggested by a VPL study involving sleep deprivation.^29^

An interesting possibility is that posttraining phenomena in VPL previously considered to be unrelated to one another^4^^,^^5^^,^^6^^,^^7^^,^^8^^,^^9^^,^^10^ could be linked by these reversal dynamics.

In perceptual deterioration, performance increases with an increasing amount of practice on a visual task, followed by a performance decrease after the amount of practice exceeds a certain level within a session.^5^^,^^6^^,^^7^^,^^8^^,^^9^^,^^10^ Perceptual deterioration may be at least partially regarded as a manifestation of the reversal dynamics suggested above. Indirect evidence that shows an association between perceptual deterioration, decreased blood-oxygen-related dependent (BOLD) signals^30^ and, in turn, decreased cortical excitation^31^ gives additional credence to this possibility.

Shibata et al.^4^ found that training on a visual task beyond asymptotic performance increases (overlearning) rapidly changed E/I ratios from higher to lower levels compared to pretraining baselines. A higher E/I ratio has been found to be correlated with a larger amplitude of VPL,^32^^,^^33^ which is also associated with higher cortical excitation.^21^^,^^34^^,^^35^^,^^36^^,^^37^^,^^38^ Therefore, the rapid decrease in the E/I ratio after overlearning may be related to the reversal dynamics. In the Shibata et al. study,^4^ no significant difference was found between the performance before overlearning training on the same day and that on the following day. In our results, performance impairments disappeared by the following day. Thus, results of both the overlearning study and this study are consistent in that intervention effects were not observed on the following day. However, in the overlearning study, performance was not measured after overlearning training on the same day. Thus, in a future study, it is necessary to test whether a temporary performance decrease did in fact occur on the same day after the overlearning training.

In summary, we found that H-RVS increased performance without prior training, whereas H-RVS severely impaired performance due to preceding visual training. These results may be a manifestation of reversal dynamics in which conditions of increased overall excitation in networks lead to a decrease instead of a further increase of signal-related activity in visual networks. This principle would be of substantial importance to rehabilitative^39^ and augmented cognition applications^40^^,^^41^ that make use of sensory stimulation protocols such as H-RVS.

### Limitations of the study

The impairment effect after training-based VPL reported here, though strong, largely relies on a subset of subjects in the H-RVS group. Arguably, only five out of twelve subjects were susceptible to this impairment (corroborated by a k-means cluster analysis). Further research would need to address the reasons why such large interindividual differences exist.^42^^,^^43^^,^^44^^,^^45^ Though there was some degree of performance differences prior to H-RVS, these did not have a discernible effect on post-stimulation performance according to our mediation analysis.

## STAR★Methods

### Key resources table

**Table:** 

REAGENT or RESOURCE	SOURCE	IDENTIFIER
**Software and algorithms**		

MATLAB Version R2019b (data analysis), Version 2012b (psychophysics)	The MathWorks, Inc.	https://www.mathworks.com
Psychophysics Toolbox, Version 3	Brainard,^46^ Kleiner et al.^47^	https://psychtoolbox.org
JASP, Version 0.11.1	JASP Team^48^	https://jasp-stats.org

### Resource availability

#### Lead contact

Further information and requests for resources should be directed to and will be fulfilled by the lead contact, Takeo Watanabe (takeo_watanabe@brown.edu).

#### Materials availability

This study did not generate new unique reagents.

### Experimental model and subject details

We recruited 61 naive subjects, of which 5 had to be excluded from subsequent analysis (see below). The remaining 56 subjects (34 female) had a mean age of 25.1 ± 1.2 years (range: 18–60 years). The subjects did not suffer from neurological/psychological conditions and had normal or corrected-to-normal vision. All subjects gave written, informed consent in accordance with institutional regulations and the Declaration of Helsinki. The subjects were randomly assigned to different experimental groups. There was no discernible influence of sex on the results of this study.

### Method details

#### Environment & apparatus

All experiments were performed in a dimly lit room (<0.5 cd/m^2^). Subjects placed their heads on an adjustable-height chin rest in front of a computer screen. In the preliminary experiment, stimuli were presented on an I-O DATA LCD-GC251USB LCD running at a resolution of 1920 × 1080 pixels and a refresh rate of 240 Hz, with 50 cm of viewing distance. In the main experiment, stimuli were presented on a Dell Trinitron Ultra-Scan P991 CRT monitor, running at a resolution of 1024 × 768 pixels and a refresh rate of 120 Hz, with 70 cm of viewing distance. Gamma corrections were performed to linearize the luminance curve. All stimuli were generated and presented using custom software written for MATLAB R2012b (The MathWorks Inc., Natick, MA) and the Psychophysics Toolbox 3.^46^^,^^47^ The stimuli were presented on a uniform gray background (62 cd/m^2^). Before and during stimulus presentation, a fixation dot of 0.1° diameter and 37.2 cd/m^2^ luminance was presented in the center of the screen. The subjects were instructed to keep their gaze on the fixation dot.

#### Orientation detection task

We employed a two-interval-forced-choice (2IFC) orientation detection task to assess the behavioral effects of RVS (Figure 1D). Orientation detection was chosen over discrimination to be able to align stimulus orientation during both task and RVS (cf.^14^). In each trial, the subjects had to indicate which of two successively presented noise fields contained an embedded Gabor stimulus as a signal image. Difficulty of this task was varied by changing the signal-to-noise level of the Gabor stimulus (i.e., percentage of signal pixels through substitution) by a 3-down/1-up staircase procedure. To advance quickly to an adequate difficulty level, the procedure was changed to a 1-down/1-up at the beginning of a block until the first mistake was made. With each step up or down, the signal-to-noise percentage was changed by 0.0612 log-levels. The initial signal-to-noise level in each block was 32.4%. Signal and noise images were shown in a circular aperture of 5° diameter in the center of the screen. The target Gabor stimulus orientation could be either of two oblique orientations (−45° or +45°) within one block. Spatial frequency was kept constant at 2 cycles per degree, while phase was randomized from trial to trial. The maximum Michelson contrast between troughs (49.6 cd/m^2^) and peaks (74.4 cd/m^2^) of the sine gratings was 0.2. The Gaussian envelope of the Gabor had a σ of 1°. Noise pixels were drawn from a Gaussian luminance distribution (μ = 62 cd/m^2^, σ = 12.4 cd/m^2^). To avoid local contrast cues unrelated to orientation detection, we ensured that local contrast in the noise images was kept similar to that in the signal images. We did this by generating noise images by randomly shuffling pixels from the signal image within concentric rings. In each trial, the prestimulus interval lasted for 300 ms. The stimulus and noise images were presented for 50 ms each (with their order randomly varied), with a 300-ms interstimulus interval. The subjects had to respond within 1.5 s by pressing ‘1’ or ‘2’ on a keyboard to indicate the interval containing the signal image. Before the start of the experiment, the subjects were instructed to respond as accurately and fast as possible. In the preliminary experiment, no feedback was presented in order to reduce the amount of training-based VPL. In the main experiment, feedback was provided by visual cues after each trial: a ‘☺’ glyph after a correct response and a ‘×’ glyph after an incorrect response. Additionally, a beeping sound was played if the subject did not respond in time. The subjects performed 16 blocks per session of 100 trials each. In the session 90 min after RVS, the subjects completed 32 blocks to better track the time-course of the effect of RVS on performance. Of these 32 blocks, only the first 16 were analyzed for comparability. The target Gabor orientation was alternated between blocks (Figure 1B). The order (i.e., −45° or +45° first) was kept consistent for each subject but was randomly varied between subjects. A cue stimulus informed subjects of the target orientation before each block.

#### Repetitive visual stimulation protocol

We tested the effects of three different repetitive visual stimulation paradigms: H-RVS, L-RVS and no-RVS. In the H-RVS and L-RVS groups, a Gabor stimulus was flickered on and off in protocol-specific temporal patterns (Figure 1E; see below). The orientation of the Gabor was either −45° or +45° and was randomly chosen for each subject. The Gabor stimuli had the same parameters as in the orientation detection task except for contrast and a lack of embedded noise. The maximum Michelson contrast at the center of the Gabor was 0.6, the luminance varied between 99.2 cd/m^2^ at the peaks and 24.8 cd/m^2^ at the troughs of the sine grating. During H-RVS, these Gabor stimuli rapidly flickered on and off in an intermittent fashion. In each cycle, the stimuli flickered on at a rate of 10 Hz and remained visible for 8.3 ms over a duration of 1 s (Figure 1E). Thereafter, no stimuli were presented for 5 s until the next cycle started. Cycles were repeated 600 times without interruption, which took 60 min. The frequency of 10 Hz was chosen over the 20 Hz used in our previous study^14^ because of the small effect sizes found in that study and the greater preponderance of H-RVS frequencies close to 10 Hz in earlier studies,^11^^,^^12^^,^^13^ suggesting that this frequency may lead to a more robust effect. In the case of L-RVS, the stimuli flickered at a low, continuous frequency of 1 Hz (Figure 1E). A cycle in this case consisted of just one Gabor stimulus being displayed for 8.3 ms until the next cycle started after 1 s. This was repeated for 3600 cycles to match the total duration of H-RVS.

To ensure compliance with central fixation, we employed an oddball task where subjects had to detect infrequent contrast changes in the Gabor stimuli. Unlike in some earlier experiments,^14^^,^^15^ this did not render the stimulus task-irrelevant, which may subject it to attentional inhibition and bias results.^1^^,^^49^ In the case of H-RVS, oddball stimulus trains had a maximum contrast of 0.9 (sine peaks: 117.8 cd/m^2^; troughs: 6.2 cd/m^2^) and 0.98 during L-RVS for single oddball stimuli (sine peaks: 122.8 cd/m^2^; troughs: 1.2 cd/m^2^). The number of contrast changes was 50 for both H-RVS and L-RVS.

Only a fixation dot (0.3° diameter) was presented in the no-RVS group, which also lasted for 60 min. During the fixation task in the no-RVS group, color of the fixation dot occasionally changed from white to red (70% red, 10% green and 10% blue color channels) and returned to white after 8.3 ms. The number of color changes was 50, the same as in the RVS groups.

In all three RVS/no-RVS conditions, the subjects had to press the space bar on a keyboard within 800 ms after a change in the target stimuli was detected. They received audiovisual feedback, as in the orientation detection task, on hits, misses and false alarms.

#### Experimental procedure

Preliminary experiment (Figure 1A): In the pretest (“pre”), subjects performed the orientation detection task for 16 blocks that lasted for approximately 45 min. The subjects could take short breaks between blocks. Immediately thereafter, subjects received 1 h of H-RVS or no intervention at all. After a break of 90 min, subjects performed the orientation detection task for 32 blocks as the posttest (“post”).

Main experiment (Figure 1C): During the first seven days, the subjects performed 16 blocks of the orientation detection task per day, which lasted for approximately 45–50 min. The subjects could take short breaks between blocks. On day eight, a pretest of the orientation detection task for 16 blocks (“pre”) preceded the 1-h session of either H-RVS, L-RVS or no-RVS for a duration of 1 h. After a break of 90 min to ensure time for neuroplastic processes to take hold and for early post-stimulation adaptation effects to subside, a posttest of the orientation detection task was performed for 32 blocks (“post1”). On the next day, one final session was performed for 16 blocks to track the persistence of RVS effects to the next day (“post2”).

### Quantification and statistical analysis

#### Statistical analysis

For statistical analysis of the behavioral data, we used JASP 0.11.1^48^ (JASP Team) and custom scripts written in MATLAB R2019b (The MathWorks Inc., Natick, MA). Unless noted otherwise, statistical tests were two-tailed where applicable.

#### Orientation detection data

During the orientation detection task, we evaluated signal-to-noise thresholds with a 3-down/1-up staircase procedure, which nominally converges on a 79.4% correct response rate.^50^^,^^51^ We calculated the geometric mean of the last 6 reversals of each block as the threshold. Likewise, we used the geometric mean for session and group means. For purposes of statistical inference, thresholds were then log-transformed. In the correlation analyses (Figures 4A–4D), we calculated Kendall’s τ_B_ as correlation coefficient to reduce the outsized influence of outliers in some analyses. For block-wise analysis in the first post session of the main experiment, we fitted power functions of the form $y=\hat{a}x^{\hat{b}}+\hat{c}$, with x designating the experimental session and designating the mean signal-to-noise threshold. Parameters could vary without constraint. Initial values for parameter estimation were set to ${\hat{a}}_{init}=1$, ${\hat{b}}_{init}=0$ and ${\hat{c}}_{init}=M_{geom}\left(y\right)$ (i.e., a constant function at the geometric mean of pre-stimulation thresholds). PL training data to learning curves was performed using the nonlinear least squares method. Parameters for a power function of the form $y=\hat{a}x^{\hat{b}}+\hat{c}$ (with constraints: $\hat{a},\hat{c}\geq 0$ and $\hat{b}\leq 0$) were estimated for each individual subject (collapsed over orientations), with x designating the experimental session and y designating the mean signal-to-noise threshold. Parameter constraints were used as VPL was expected to occur. Initial values for parameter estimation were set to ${\hat{a}}_{init}=1$, ${\hat{b}}_{init}=0$ and ${\hat{c}}_{init}=M_{geom}\left(y\right)$. Posttraining slopes of the learning curves were obtained by differentiating each fitted function at x=7.

#### Repetitive visual stimulation

As a means of fixation compliance during all types of RVS, we calculated d’ to estimate how well subjects paid attention to infrequent changes in the target stimuli. Successful responses within 800 ms after the changes were counted as hits, while responses outside of this time window were counted as false alarms. The subjects in the no-RVS group, who were not presented with oriented Gabor stimuli, were assigned an arbitrary orientation (−45°) as the ‘stimulated’ orientation for later analysis. Both hit and false alarm rates were bounded at .02 and .98 for obtaining finite d’ values in all cases. The fixation compliance during RVS did not significantly correlate with subsequent impairment (not shown).

#### Exclusion criteria

Some subjects did not approach stable performance in the orientation detection task but instead showed severe impairment at some point in the training procedure (but before RVS) of the main experiment. Therefore, we decided to exclude subjects who demonstrated this pattern, which was likely related to motivational instead of perceptual issues. We thus excluded 5 subjects from the analysis whose (log-transformed) pre-RVS threshold on day 8 was between 75 and 300% worse than their best daily mean threshold during the training phase (at most 16% worse for all others).

#### Mediation analysis

In the mediation analysis, standard errors were calculated using the delta method. We used a maximum-likelihood estimator and the bias-corrected percentile bootstrap method (B = 1000) for confidence interval estimation. Estimated model coefficients were standardized. As the categorical predictor variable of the model had to be binary, we recoded subjects according to whether they received H-RVS or not (L-RVS or no-RVS), instead of using group as the predictor variable.

## Data Availability

• All data reported in this paper will be shared by the lead contact upon request. • This paper does not report original code. • Any additional information required to reanalyze the data reported in this paper is available from the lead contact upon request.
